# A Rare Complication of Pneumopericardium, Spontaneous Pneumothorax, and Subcutaneous Emphysema in a COVID-19 Pneumonia Patient Treated With High Flow Nasal Cannula

**DOI:** 10.7759/cureus.24795

**Published:** 2022-05-07

**Authors:** Mei L Tan, George B Thomas

**Affiliations:** 1 General Medicine, Khoo Teck Puat Hospital, Singapore, SGP

**Keywords:** covid-19, sar-cov-2, simple pneumopericardium, post covid-19 complication, post-acute covid-19 syndrome, spontaneous pneumomediastinum (spm)

## Abstract

Spontaneous pneumomediastinum (SPM), unrelated to mechanical ventilation, has been newly described as a complication of patients with coronavirus disease (COVID-19) pneumonia without the associated risk factors. The main objective of presenting this case is to highlight a rare but important complication among patients with COVID-19 pneumonia treated only with a high-flow nasal cannula (HFNC). We aim to study the possible underlying pathophysiology of this phenomenon.

## Introduction

Spontaneous pneumomediastinum (SPM) is a rare and self-limiting condition characterized by the presence of air in the mediastinum not related to trauma or surgical procedures [1]. Described by Laennec in 1819 as a complication of trauma, Hamman 120 years later published his case series of SPM. This condition typically affects young adults aged 20-30 years, with a male preponderance of 8:1. SPM is associated with other medical conditions, including asthma, connective tissue disease, interstitial lung disease, diabetic ketoacidosis, chronic obstructive airway disease, and influenza-like syndrome. [1] SPM is reported to develop in 10% of cases of intubated COVID-19 with acute respiratory distress syndrome (ARDS) even with low tidal volume strategies [2,3]. One unmatched case control study of 271 patients showed the incidence of SPM among non-intubated acute COVID-19 patients at 3.3%, similar to our patient [4]. The case we described would fit into this group. 

The cause of COVID-19, SARS-CoV 2, is a novel coronavirus associated with wide heterogeneity in clinical presentation ranging from asymptomatic to critical illness. The first infection was detected in late 2019 in Wuhan, China, after which it rapidly spread worldwide. Mortality was high among those with advanced age and significant comorbidities. The acute phase of COVID-19 infection lasts approximately three to four weeks. After four weeks of infection, SARS-CoV 2 no longer has the capability to replicate, and residual illness in this stage is called the post-acute COVID-19 syndrome [5]. Symptoms associated with the infection may persist, such as lethargy, easy fatigability, and shortness of breath, with some requiring prolonged supplemental oxygenation. To our knowledge, the incidence and risk factors of SPM among patients who have recovered from COVID-19 infection, i.e., patients in the post-acute phase, is yet to be studied.

We present a case of SPM in a patient with post-acute COVID-19 syndrome who received high flow nasal oxygen therapy in the acute stages of the disease.

## Case presentation

The patient was a 58-year-old Chinese gentleman who never smoked. He had a BMI of 29.4kg/m2 and was fully vaccinated for COVID-19 (last dose was given three months prior to admission). He was admitted to the emergency room complaining of a productive cough accompanied by shortness of breath. A nasal pharyngeal swab for polymerase chain reaction (PCR) detecting SARS‐CoV‐2 ribonucleic acid (RNA) resulted in a positive. His significant medical history included hypertension and hyperlipidemia. He had no prior trauma, asthma history, diabetes, pulmonary tuberculosis, or connective tissue disease.

**Figure 1:**
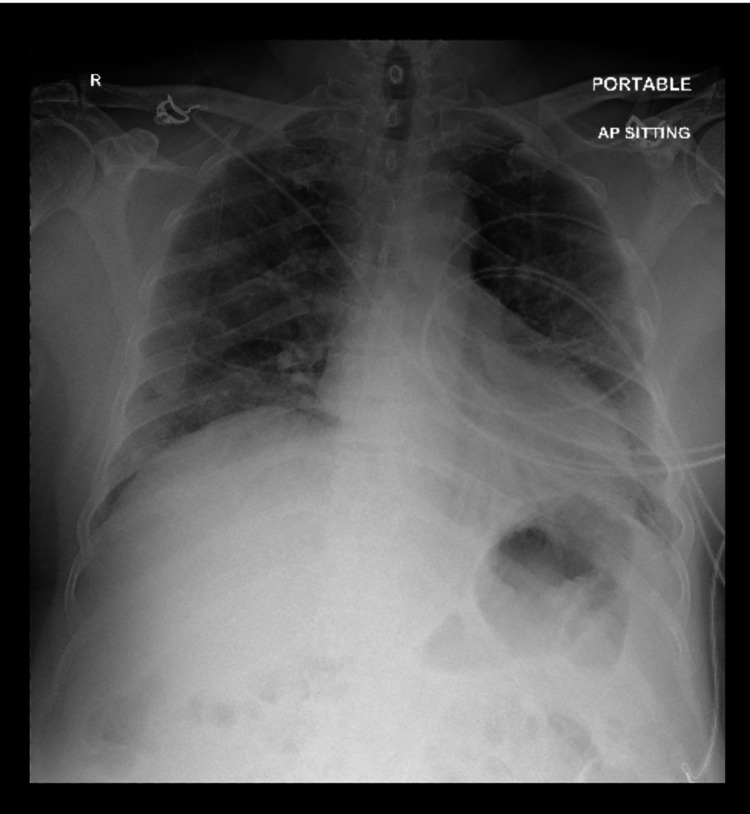
Chest X-Ray showing right middle and lower zone patchy airspace opacities without pleural effusion or pneumothorax

On admission, physical examination showed decreased breath sounds on both lungs and diffuse systolic murmur. He was febrile at 38 degrees Celsius, with a blood pressure of 114/77mmHg, heart rate of 143 per minute, and respiratory rate of 35 per minute. Oxygen saturation was at 89% on 100% non-rebreather mask. Arterial blood gas showed type 1 respiratory failure with a P/F ratio of 46. Therefore, we decided to start oxygen therapy with a high-flow nasal cannula (HFNC) (FiO2: 100%, flow: 60 L/min with SpO2: 96%). Initial chest X-ray (CXR) revealed right middle and lower zone patchy airspace opacities without pleural effusion or pneumothorax (Figure 1).

The full blood count showed a white blood cell count (WBC) of 6.95x10^9/L, hemoglobin 15.2g/dL, platelets 191x10^9/L, C-Reactive protein (CRP) 151.1mg/L (N=1.0-5.0mg/L), procalcitonin 1.2ng/mL (N=0.5 to 2.0ng/mL), serum lactate 1.9mmol/L (N=0.6-1.4mmol/L), serum urea 6.8mmol/L (N=2.4 to 6.6mmol/L) and beta-hydroxybutyrate 0.35mmol/L (N=0.02-0.27). His International Severe Acute Respiratory and Emerging Infection Consortium (ISARIC) 4C score was nine, signifying high risk and an in-hospital mortality of 31.4 to 34.9%. The patient was prescribed intravenous dexamethasone before transferring to medical intensive care unit (MICU).

In the medical intensive care unit, he received empiric intravenous amoxicillin-clavulanic acid and oral doxycycline. Blood cultures and sputum cultures, which were taken from endotracheal tube (ETT), were all reported as no bacterial growths. Fever persisted, and a repeat chest X-ray showed worsening bilateral airspace opacities. Antibiotics were escalated to intravenous piperacillin-tazobactam while on intravenous dexamethasone therapy. Blood cultures and sputum cultures were repeated and were negative. On day six of illness, he received the first dose of a five-day course of intravenous remdesivir. Due to persistent hypoxia, he received two doses of intravenous tocilizumab on day six and day 26 of illness.

He also developed starvation ketosis and revealed newly diagnosed diabetes mellitus with HbA1c of 8.1%. Subcutaneous (SC) intermediate-acting insulin (Insulatard) was prescribed. Thromboprophylaxis with subcutaneous enoxaparin was given during the pulmonary phase of the illness. The HFNC setting for the first 15 days was on maximum flow of 60L/min, with a taper to 40L/min on the remaining seven days. Initial FiO2 on HFNC was at 100%, with subsequent gradual weaning to 40% on day 28 of illness. On day 10 of illness, he received a trial of continuous positive airway pressure ventilation (CPAP), but HFNC was resumed as no significant improvement was seen on oxygenation. CRP levels improved from 151mg/L to 72.8mg/L, and by day 28 of illness, the CRP was at 4.4mg/L. The patient performed awake prone positioning to improve oxygenation.

On day 30 of illness, he was weaned off to a non-rebreather mask and managed to sustain adequate oxygen saturation on nasal cannula oxygenation at 5-liter oxygen. He was transferred to the general ward for rehabilitation. He remained afebrile and normotensive with a resting tachycardia at 100-110/min. Despite mild dyspnea and easy fatigability, oxygen saturations were at 98% on 4-liter oxygen nasal cannula. Intravenous dexamethasone was gradually tapered down.

**Figure 2:**
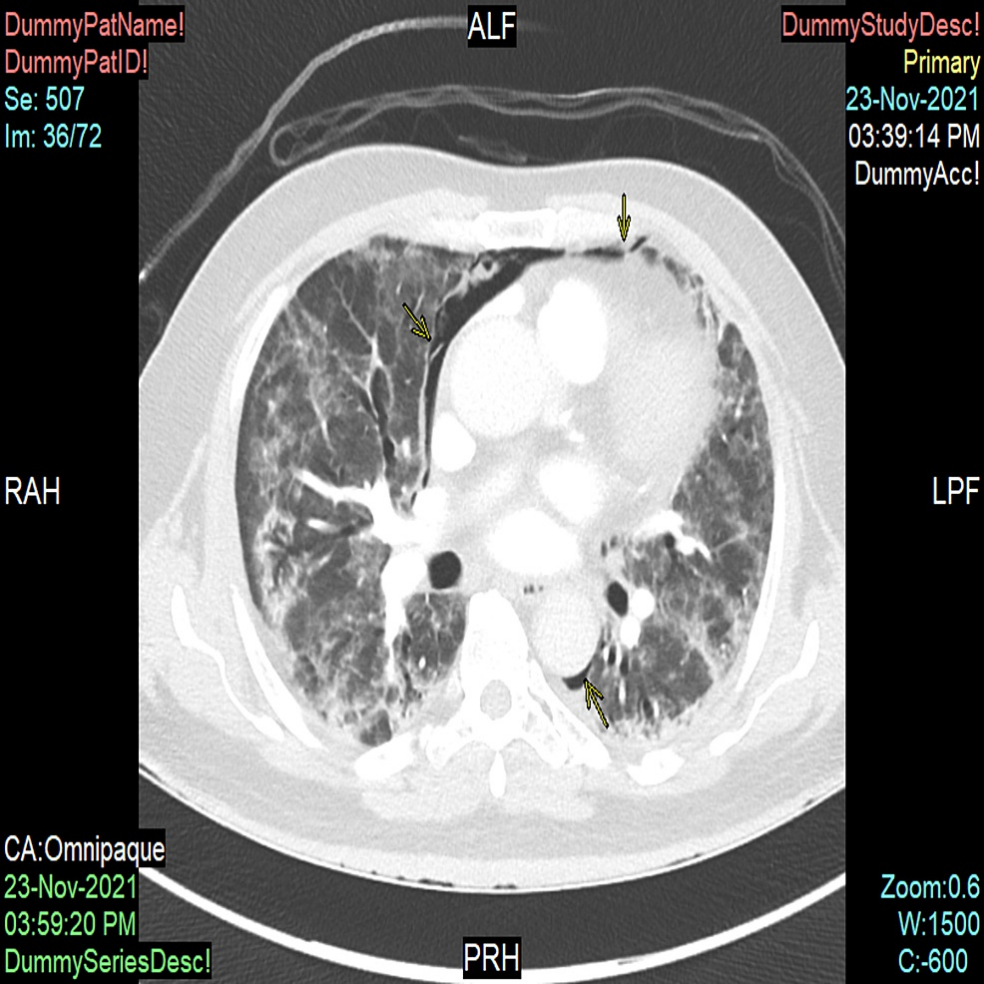
Computed tomography scan in axial view of the patient demonstrating pneumomediastinum (yellow arrows indicating the layering of air alongside pulmonary vasculature)

**Figure 3:**
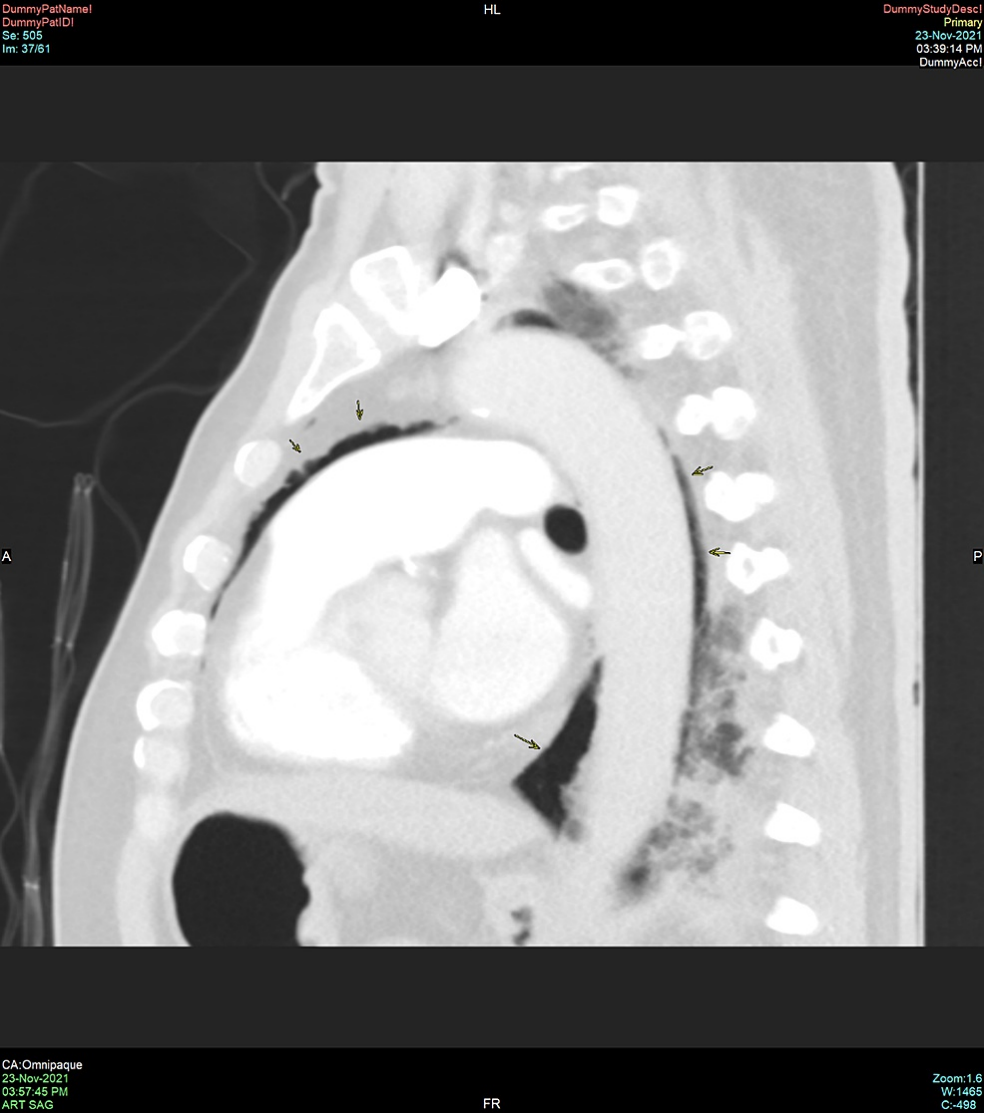
Sagittal view of a computed tomography scan of the thorax demonstrating pneumomediastinum (black arrows)

On day 34 of illness, COVID-19 PCR with cycle threshold (CT) ratio was 33.34/33.37. Despite this, his saturations dropped to 77% while on 4-liter oxygen nasal cannula. He was put on 100% non-rebreather mask, and oxygen saturations increased to 96-99%. Repeat CXR showed stable bilateral diffuse airspace opacities with no evidence of pneumothorax. Repeat arterial blood gas revealed type 1 respiratory failure with a P/F ratio of 119 and CRP of 0.7mg/L. An electrocardiogram showed normal sinus rhythm at 73/min. A computed tomography pulmonary angiogram (CTPA) was arranged to rule out acute pulmonary embolism. SC enoxaparin was restarted at a therapeutic dose. The scan was negative for pulmonary embolism but detected a pneumomediastinum (PM), pneumopericardium (PP), and subcutaneous emphysema (Figure 2, 3). Respiratory medicine service recommended keeping him on non-rebreather mask oxygenation, and he was deemed a poor candidate for positive pressure ventilation in the event of current deterioration. On examination, he was alert tachypneic with bilateral scattered crackles in the middle and lower zones on auscultation. He developed subcutaneous emphysema at the neck but no change in the quality of his voice. After discussion with the patient and his family, he opted for maximum ward management in the event of further deterioration. The family was hopeful for his full recovery. On day 36 of illness, he developed atrial flutter with a pulse rate of 160bpm on 12-lead electrocardiography (ECG) with a blood pressure of 109/79mmHg. He received rate control measures, including intravenous amiodarone, oral bisoprolol, and digoxin, and his heart rate improved to sinus rhythm at 76bpm on 12-lead ECG.

On day 40 of illness, the patient was found unresponsive with pulseless electrical activity on the cardiac monitor. Cardiopulmonary resuscitation (CPR) was initiated and he was intubated by the on-call airway team. Despite the resuscitation team’s best efforts, no return of spontaneous circulation was achieved, and the patient was pronounced demised.

## Discussion

There are a number of mechanisms that lead to the development of spontaneous pneumomediastinum. First is the alveolar rupture secondary to inflammation and diffuse alveolar pressures due to coughing. The escaping air from the ruptured alveoli tracks along the bronchovascular sheaths, dissecting into the pulmonary hila and escaping into the mediastinal space. This is seen on thoracic computed tomography scans demonstrating the Macklin effect, described as linear collections of air continuous to the bronchovascular sheaths dissecting into the pulmonary hilum [6]. Second is the direct viral invasion of the lung parenchyma, visceral and parietal pleura causing disruption of the parenchymal and pleural integrity or ruptured alveoli leading to subsequent air leak [7]. Third is the prothrombotic effect of COVID-19 infection-causing pulmonary vascular thrombosis and subsequent necrosis in the alveolar membranes. Fourth is cytokine storm-induced diffuse alveolar injury or direct viral infection of type 1 and type 2 pneumocytes increasing the risk of alveolar rupture [3].

COVID-19 related SPM affects an older population aged 38-72 years of age versus 5-34 years for non-COVID SPM [8]. COVID-19 related SPM has been associated with a more severe course of the disease and a mortality rate of 28.5% versus non-COVID SPM, which has an estimated mortality rate of 5.6% [1].

## Conclusions

We highlight the risk of SPM, PP, and subcutaneous emphysema developing in COVID-19 patients without the usually associated conditions who did not receive invasive positive pressure ventilation at the post-acute phase of the disease. We also hope this launches further investigations comparing the non-invasive and invasive modalities of oxygen supplementation and the respective settings for severe COVID-19 to achieve the optimal oxygenation profile while minimizing the risk of barotrauma and PP and PM. We also anticipate more studies that look into developing multidisciplinary treatment protocols for patients who develop COVID-19 related PP and PM. The question is: which modality achieves optimal oxygenation while minimizing the risk of barotrauma and SPM? 
